# Knowledge and nutrition-related practices among caregivers of adolescents with sickle cell disease in the Greater Accra region of Ghana

**DOI:** 10.1186/s12889-023-15343-1

**Published:** 2023-03-06

**Authors:** Agartha Ohemeng, Eunice Berko Nartey, Esi Quaidoo, Richard Stephen Ansong, Matilda Steiner Asiedu

**Affiliations:** 1 Department of Nutrition and Food Science, School of Biological Sciences, College of Basic and Applied Sciences, University of Ghana, Legon Boundary, Accra, Ghana; 2 Department of Nutrition, School of Public Health and Health Sciences, University of Massachusetts Amherst, Amherst, MA United States of America

**Keywords:** Sickle cell disease, Nutrition-related, Caregivers, Adolescents, Ghana, Nutrition practices

## Abstract

**Background:**

Due to its severe adverse effect on child mortality, sickle cell disease (SCD) has been identified as a set of diseases of public health concern. The high mortality rate among children with SCD in Africa has been attributed to several factors including sub-optimal management and care. This study documented the nutrition-related knowledge and practices of caregivers of teenagers who suffer from sickle cell disease (SCD) to inform decisions on integrated management of the disease.

**Methods:**

The study included caregivers (n = 225) of adolescents with SCD who attended clinic at selected hospitals in Accra, Ghana. Pre-tested semi-structured questionnaire was employed in the gathering of information related to general and nutrition-related knowledge about SCD, as well as data on their nutrition-related practices with regards to their children who suffer from SCD. Pearson’s Chi-square test and binary logistic regression analyses were applied to explore the relationship between caregivers’ nutrition-related knowledge and practice.

**Results:**

Nutrition-related knowledge among the caregivers studied was low, with less than a third of them (29.3%) of the sample being classified as having good knowledge. Caregivers who considered nutrition care when the child experienced crises were few (21.8%), and those with low nutrition-related knowledge were less likely to do this compared with caregivers having high knowledge (OR = 0.37, 95% CI = 0.18, 0.78). The common nutrition actions reported were the provision of more fruits/fruit juices (36.5%) and warm fluids such as soups and teas (31.7%). More than a third of the caregivers (38.7%) admitted that they faced challenges in caring for their adolescents with SCD, particularly in the area of finance for the needed health care.

**Conclusion:**

Our study findings indicate that it is important to incorporate appropriate nutrition education messages for caregivers as part of a holistic management of SCD.

**Supplementary Information:**

The online version contains supplementary material available at 10.1186/s12889-023-15343-1.

## Background

Sickle Cell Disease (SCD) refers to a set of congenital haematological disorders that cause bone marrow to produce abnormal red blood cells (RBCs) [1]. These abnormal RBCs cause occlusions to regular blood flow and can be a source of excruciating pain [2]. SCD is a haematological non-communicable disease (NCD), sometimes referred to as the invisible NCD [2]. It is a public health problem that affects around 5% (includes sickle cell trait and sickle cell disease) of the world’s population [3], with the highest prevalence found in sub-Saharan Africa[4, 5]. It is estimated that 15,000 babies are born with this condition every year in Ghana alone [6]. Manifestations of SCD include hyper-metabolism, infections, bone/joint necrosis, painful crises, and sometimes exacerbation of chronic anaemia. SCD can also cause organ damage and premature death due to preventable complications [7, 8]. Sickle cell anaemia alone contributes about 16% in some West African countries and 5% of under five deaths in Africa [9]. In view of these facts, WHO resolved to increase awareness of the condition, promote equitable access of SCD patients to health services, and promote and support research to improve quality of life for those affected [10].

Bone marrow and stem cell transplants are promising treatments that remain costly in developing countries, with many caretakers of affected children lacking the means to provide their wards with such options [11]. There are, however, strategies that aim to reduce mortality and morbidity due to SCD and these include both supportive and preventive management. The high mortality rate among children with SCD in Africa has been attributed to several factors including sub-optimal management and care. In discussing the management options and challenges of SCD in developing countries, experts [5, 12, 13] have advocated that emphasis should be placed on early counselling, new-born screening, anti-microbial prophylaxis, vaccination against infections, and training of healthcare workers, patients, and caregivers. These strategies are relatively cheaper and can be implemented through existing health care systems, once the political and financial commitment is present.

Studies in various settings have indicated that children with SCD tend to present sub-optimal nutrition [14–17], which in turn adversely affects SCD severity, quality of life, and survival. The observed malnutrition is probably due to hypermetabolism, altered metabolic pathways, and lower food intakes that are associated with the disease. In Ghana, few available studies have indicated that dietary intakes of children with SCD are woefully inadequate with respect to most micronutrients [15, 18, 19]. Additionally, children with genotype HbSS had a lower tendency to meet daily requirements. Thus, the need to develop a comprehensive management strategy for SCD patients that intentionally includes nutritional therapy is urgently needed. Studies have shown that nutrient supplementation can have a positive impact on the overall health of individuals with SCD [17, 20, 21]. Nutritional therapy should include nutrition education and counselling, as well as supplements supported by empirical evidence. This is because knowledge on food selection and appropriate combinations among others is critical and does influence dietary practices and ultimately nutritional status [20]. It is evident that healthy nutritional status is fundamental in improving the quality of life of individuals with SCD, therefore mobilizing efforts to improve awareness of the best nutrition practices is necessary.

Caregivers in general provide physical and emotional support for their wards. Providing care for a child or individual with a chronic disease such as SCD can be quite daunting, and impairments in the wellbeing of caregivers can influence the quality of care received by children with SCD [22]. Studies have shown that primary caregivers of SCD children have higher levels of depression compared to caregivers of their healthy counterparts [23, 24]. The studies that have explored SCD caregivers’ perspectives have documented premarital SCD-status knowledge, caregiver action plans during SCD crises, caregiver-SCD-ward relationship dynamics, caregivers’ mental health, coping mechanisms, and burdens of responsibility [4, 25, 26]. Thus, most of the existing research on the capacity of caregivers of SCD children to provide the needed care have concentrated on the psychological domain. The UNICEF Conceptual Framework emphasizes diets and care as immediate determinants of child nutrition [27], and these have practices and services as underlying factors/determinants in addition to the food itself. Thus, the kind/level of caregiving is also influenced by the qualities of the caregiver such as their knowledge, practices, and social settings. While the pool of published literature on SCD is substantial, there is a paucity of literature from the sub-Saharan context on the SCD knowledge, SCD nutrition-related attitudes and practices of individuals who are tasked to care for children with this condition. Evaluation of the awareness of health issues that affect a huge portion of the population is important as it can provide empirical evidence that can guide public health interventions and inform community-specific health policies [28, 29]. Therefore, the primary aim of this study was to assess the basic knowledge on SCD and nutrition of caregivers of adolescents with SCD, document their practices in caring for their wards, and examine their nutrition related attitudes.

## Methods

### Study design and area

This was a cross-sectional study that assessed the knowledge, attitudes, and nutrition-related behaviours of primary caregivers of adolescents with Sickle Cell Disease (SCD). Data were collected at the Sickle Cell Disease Clinics (SCDC) of four public hospitals located in the Greater Accra Region. The study was conducted from October 2019 through to February 2020.

### Study Population, Sample size, and Sampling

The study population consisted of primary caregivers whose wards were aged 10–19 years and diagnosed with SCD. Sample size was calculated to be 206, based on the proportion of tertiary students who were classified as having good knowledge about SCD [30], 7% precision and 95% confidence interval. Within the study period, the research team interacted with all caregivers who visited the study hospitals with their wards on sickle cell clinic days and who met the eligibility criteria (i.e. be the primary caregiver of a child with SCD aged 10–19 years). All the caregivers who were approached by the research team agreed to participate in the study. Most caregivers were more willing to be interviewed whilst waiting for medical care at the hospital as compared to phone interviews away from the hospitals. A total of 225 caregivers of adolescents with SCD participated in the study.

### Ethics approval

This study was part of a larger study that sought to provide a nutrition education intervention to teenagers with SCD and this trial was registered with the International Standard Randomised Controlled Trial Number (ISRCTN) with registration number ISRCTN17054215. All procedures of this study were conducted according to the guidelines and regulations laid down in the Declaration of Helsinki. The parent study was approved by the Ethics Committee of the College of Basic and Applied Sciences, University of Ghana (Study ID: ECBAS 025/17–18). Thus, all procedures followed for this study was approved by the Ethics Committee of the College of Basic and Applied Sciences, University of Ghana. Official approval was sought from the hospital administrations of all four hospitals before the research team began collecting data on hospital premises. The objective of the study was explained to potential study participants and voluntary written informed consent was obtained before data collection was done.

### Data collection procedures

The questionnaire developed for this study was interviewer-administered, lasting for approximately 25 min, excluding the data on sociodemographic and clinical history. The study questionnaire was pre-tested in another health facility before the study.

#### Sociodemographic and clinical information

The team collected sociodemographic information about the primary caregivers (age, ethnicity, gender, religion, occupation, attained highest formal education, relation to ward), as well as clinical information about the index children (age of SCD diagnosis, presence of other medical conditions, hospitalisation in the past six months). Information was also gathered on the frequency of crisis episodes experienced by the ward, actions taken during these episodes, and the medications the ward was currently on. Caretakers were also asked whether they considered any sort of nutritional care for their ward during crises.

#### Knowledge assessment

In assessing the caregivers’ basic knowledge of SCD, respondents were asked how SCD is contracted, how SCD is diagnosed, the lifespan of a sickle red blood cell, major signs, and symptoms of SCD, and the probability of a child inheriting SCD given both parents have the sickle cell trait. Five questions were posed to assess knowledge on nutritional care for individuals with SCD. Caretakers were asked to identify micronutrients responsible for blood formation and food item(s) that improve iron absorption.

#### Attitudes assessment

Participants were asked to either agree or disagree with some statements using a Likert scale with options: ‘strongly agree,’ ‘agree,’ ‘disagree,’ and ‘strongly disagree’. These statements included the following: ‘*it is easy talking to friends and family about ward’s condition,’ ‘SCD is a curse from God,’ ‘pre-marital blood screening is necessary’, and ‘food can help improve the health of people with SCD’*.

#### Practices assessment

Caregivers were asked about their perceptions of the dietary habits and nutritional status of their children with SCD, and the measures they took to ensure that their wards eat well. Respondents also provided information on the challenges they face in caring for their adolescent children who suffer from SCD. The interview also assessed knowledge and utilisation of any special foods believed to improve the health of children with SCD.

### Statistical analysis

Statistical package for social scientists (SPSS) 23.0 software was used to analyse all data at 95% confidence interval. To analyse the knowledge assessment section, a scoresheet was set up where one point (1) was assigned to a correct response and zero (0) to an incorrect response. Scores for the ten questions were summed up to represent the general knowledge score. The lowest possible score was zero (0) and the highest possible score was ten (10). Caregivers with scores at and below the group mean score were categorized as having ‘poor knowledge of SCD’ and those with total score above the mean were categorized as having ‘good knowledge of SCD.” Nutrition-related knowledge score was calculated in the same way, based on only the five questions related to nutrition.

To assess the relationship between sociodemographic factors and caregivers’ level of knowledge, Pearson’s Chi-square test and binary logistic regression analyses were conducted. Similar analyses were applied in exploring the relationship between caregivers’ knowledge and the reported frequency of crisis episodes.

## Results

**Table 1:** Background characteristics of study participants (n = 225)

Characteristic	Mean or Frequency	SD or %
**Children**		
Age (years): Mean ± **SD**	14.1	± 2.8
Sex:		
Male Female	102 123	45.3 54.7
School attendance:		
Yes No	222 3	98.7 1.3
Age at diagnosis (years): Mean ± **SD**	2.1	± 2.2
Frequency of crisis episodes:		
Very often Sometimes Once a year	70 110 43	31.1 48.9 19.0
Occurrence of crisis episode:		
> Two weeks prior to survey ≤ Two weeks prior to survey	188 37	83.6 16.4
Hospital admission past 6 months:	113	50.2
**Caregivers**		
Age (years): Mean ± **SD**	41.7	± 7.7
Sex:		
Male Female	42 183	18.7 81.3
Formal Education:		
Above Secondary Secondary Basic* None	57 83 73 12	25.3 36.9 32.4 5.3
Primary occupation:		
Self-employed^a^ Government employee Other^b^ Unemployed	166 29 16 14	73.8 12.9 7.1 6.2
Relation to study child:		
Parent Other relative	204 21	90.7 9.3

^a^Includes traders. ^b^Other occupations included sales representative, textile designer, seamstress, driver, hairdresser, restaurant attendant, receptionist, security man, insurance agent. *Includes Primary and Junior High levels. Values are presented as frequencies (%) or means ± standard deviation

**Fig. 1:**
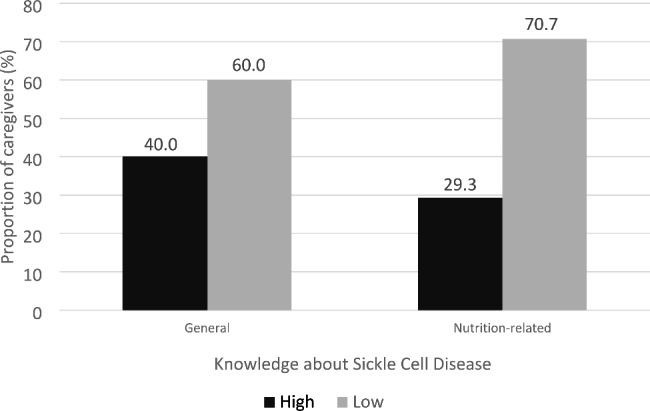
Knowledge about sickle cell disease among caregivers of teenagers with disease Fig. 1 shows the level of general knowledge as well as nutrition-related knowledge about sickle cell disease among the caregivers interviewed. The black shade represents proportion of caregivers who were classified as having high knowledge, and the grey shade represents proportion of caregivers classified as having low knowledge

A total of 225 primary caregivers of adolescents with sickle cell disease participated in this study (Table 1), with most of them being females (81.3%). The mean age of the caregivers was 41.7 ± 7.7 years and that of the index children was 14.1 ± 2.8 years. More than half of the caregivers had received at least secondary level formal education (62.2%) and majority (93.8%) had some form of employment and were parents (96.5%) of the adolescents with SCD. The mean age at which these adolescents were diagnosed with sickle cell was 2.1 ± 2.2 years. Clinical information showed that majority of the adolescents (80.0%) experienced more than one crisis episode per year (Table 1), although most of them (82.7%) had not experienced any crisis episode two weeks prior to the interviews. More than a third of the caregivers (38.7%) admitted that there were challenges in caring for their adolescents with SCD. The main challenge was financial difficulty (Fig. 1) in meeting the medical needs of the child such as purchasing drugs and paying for medical care.

Although majority of the caregivers (87.6%) interviewed agreed that food can help improve the health of people with sickle cell disease (Table 2), less than a quarter (21.8%) considered nutrition care when the child experienced a crisis. The common nutrition actions that caregivers deliberately took during a crisis were providing the child with more fruits/fruit juices (36.5%), warm fluids such as soups and teas (31.7%), and/or dark green leafy vegetables (14.3%). A little over a third (36.4%) of the participants were not satisfied with the feeding habits of their children who have SCD, and this was similar to those who indicated that their wards were selective when it comes to the foods they eat. Of the caregivers who admitted that they had to do something for their children to eat, giving encouragement (42.9%) and providing only preferred foods (41.1%) were the most common strategies, although a few applied force. Both awareness (61%) and usage (60%) of ‘special foods’ specifically for SCD were high among the participants. However, half of those who tried indicated that those ‘special foods’ did not help improve the condition of their wards. In all, more than half of the respondents (60.9%) perceived their wards with sickle cell disease to be of good nutritional status.

**Table 2:** Attitudes and nutrition-related perceptions and practices of caregivers

	Frequency	%
Easy to talk about ward’s SCD:		
Agrees Disagrees	75 150	33.3 76.7
SCD is a curse from God:		
Disagrees	225	100.0
SCD is less known and needs awareness creation:		
Agrees Disagrees	214 11	95.1 4.9
Food can help improve help health of SCD patients:		
Agrees Disagrees	197 28	87.6 12.4
Special foods exist to help improve health of child with SCD:		
Yes No Don’t know	137 87 1	60.9 38.7 0.4
Special foods have helped improve ward’s health:		
Have helped Have not helped Have not tried Special foods	67 68 90	29.8 30.2 40.0
Eating habits of wards:		
Flexible with meals Selective with meals	145 80	64.4 35.6
Consideration of nutrition care during crisis:		
Yes No	49 176	21.8 78.2
Satisfied with ward’s eating habits:	143	63.6
Ward’s nutritional status is good:		
Agrees Disagrees	155 70	68.9 31.1
Factors contributing to ward’s current nutritional status:		
Food^a^ Good medical attention Genetic disposition On-going illness Cannot tell	95 33 30 18 49	42.2 14.7 13.3 8.0 21.8

^a^Responses includes child eats well, good diet, forcing child to eat, and picky eating. Values are presented as frequencies (%)

**Fig. 2:**
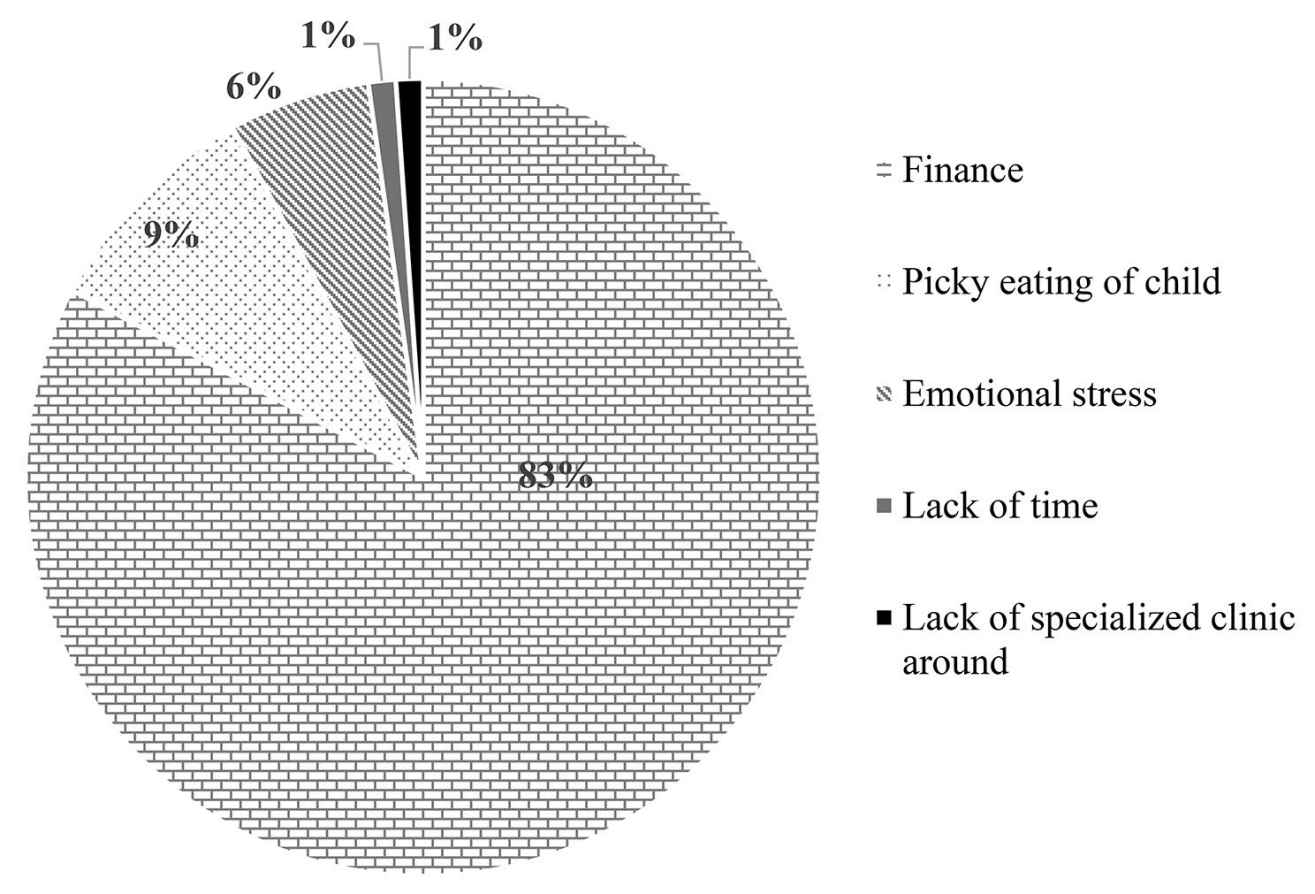
Challenges that caregivers of teenagers who have sickle cell disease experience Fig. 2 shows the different challenges that study participants identified as caregivers of adolescents with sickle cell disease. The brick layers represents those who faced financial difficulties in caring for their wards, the doted pattern represents those who identified child’s picky eating habit as a major challenge, the diagonal line pattern shows the proportion of caregivers who were emotionally stressed because of the child’s illness, grey represents those who said ‘lack of time’ was a major challenge, and black shade represents the proportion who identified ‘lack of specialized clinics within the vicinity’ as challenging in caring for a child with sickle cell disease

The general knowledge about sickle cell disease was low among the participants, with a mean score of 2.6 ± 0.1 out of a total score of 10. More than half of them (60%) were classified as having poor general knowledge (Fig. 2). It was also observed that nutrition-related knowledge was also poor, with a group mean of 1.0 ± 0.1 out of a total of five. Only about a quarter of them (29.3%) were classified as having good nutrition-related knowledge about sickle cell disease in this study. Bivariate analysis showed that the proportion of caregivers with adequate general knowledge was significantly high among those with at least secondary level formal education (Table 3). Based on a logistic regression model accounting for age and sex (Supplementary material), caregivers with at least secondary education were almost four times more likely to have good knowledge about sickle cell disease (OR = 3.90, 95% CI: 2.05, 7.40), compared to those with formal education below the secondary level. A similar trend was observed for nutrition-related knowledge. The odds of caregivers with above secondary education having a high nutrition-related knowledge was four times compared to caregivers with below secondary education (OR = 4.03, 95% CI = 1.92, 8.46).

**Table 3:** Comparison of selected nutrition-related perceptions and SCD-related crisis based on caregiver knowledge

	General SCD knowledge^a^				P-value^b^	Nutrition-related knowledge^a^				P-value^b^
	**Low** **(n = 135)**		**High** **(n = 90)**			Low (n = 159)		High (n = 66)		
Food can improve health of SCD patients					0.011					0.006
*Agree*	112	83.0	85	94.4		133	83.6	64	97.0	
*Disagree*	23	17.0	5	5.6		26	16.4	2	3.0	
Special foods exist for children with SCD:					0.001					0.005
*Yes*	71	52.6	66	74.2		88	55.3	49	75.4	
*No*	64	47.4	23	25.8		71	44.7	16	24.6	
Considers nutrition care during child’s crisis episode:	28	20.7	21	23.3	0.644	29	18.2	20	30.3	0.046
*Yes*	107	79.3	69	76.7		130	63.7	46	69.7	
*No*										
Frequency of crisis episodes:					0.003					0.015
*Often*	52	38.8	18	20.2		57	36.3	13	19.7	
*Not often*^*c*^	82	61.2	71	79.8		100	63.7	53	80.3	

^a^Level of general knowledge about sickle cell disease was categorized using the group mean as cut-off: caregivers with values above 2.6 were classified as having high knowledge, those with values below 2.6 were classified as having low knowledge. Nutrition-related knowledge was based on only the five questions related to nutrition. The total score was categorized using the group mean of 1 as cut off: caregivers with values 1 and above were classified as having high nutrition-related knowledge, those who scored zero were classified as having low knowledge.^b^Analysis based on Pearson’s chi Square test.^c^Responses include ‘sometimes’ and ‘once a year’.

A higher proportion of caregivers with high knowledge (both general and nutrition-related) agreed that food can improve the health of people with sickle cell, believed that there are special foods for persons with sickle cell disease, and reported that their wards did not experience crisis often (Table 3), compared to those with low knowledge. Accounting for age, sex and educational level, caregivers with low nutrition-related knowledge were 63% less likely (Table 4) to resort to nutritional care during crises compared with caregivers with high nutrition-related knowledge (OR = 0.37, 95% CI = 0.18, 0.78). Additionally, the odds of female caregivers resorting to nutritional care use during crises were 94% less likely compared to the male caregivers (OR = 0.06, 95% CI = 0.01, 0.43). None of the variables assessed however, significantly predicted the frequency of crises as reported by the caregivers.

**Table 4:** Association between nutritional care use during crises, frequency of crises and potential covariates, based on logistic regression model

Variable	Nutrition care during crises			Frequency of crises		
	OR	95% CI	p-value	OR	95% CI	p-value
**Age**			0.642			0.664
< 42 years ≥ 42 years	1.173 1	0.599–2.295		1.141 1	0.629–2.069	
**Sex**			0.006			0.489
Female Male	0.056 1	0.007–0.433		1.357 1	0.571–3.223	
**Level of Education**			0.564			0.296
≥ Secondary < Secondary	1.233 1	0.605–2.513		0.717 1	0.384–1.339	
**Nutrition Related Knowledge**			0.009			0.053
Low High	0.374 1	0.178–0.784		0.494 1	0.241–1.009	

Odds ratio was obtained using binary logistic regression. Hosmer – Lemeshow Statistic: p = 0.961 for the ‘nutrition care during crises’ model, p = 0.563 for the ‘frequency of crises’ model

## Discussion

Management of chronic diseases such as SCD requires knowledge of the disease and appropriate care including nutritional support. This study assessed knowledge of SCD among caregivers of adolescents with SCD as well as nutrition-related knowledge of SCD management. In this study, the mean age at which wards were diagnosed with SCD was two years old. This observation contradicts the diagnostic age reported by other authors [13, 31, 32]. For many individuals who have SCD and are resident in Sub-Saharan Africa, greater than five years old is often the diagnostic age. The lower mean age reported in this study may be due to the study location. Accra has specialized clinics for SCD, and as such diagnostic age may be lower than other parts of Sub-Saharan Africa which are comparatively resource-poor [5]. Apart from the recommendation of pre-conception genetic testing to create awareness of SC traits and to encourage informed reproductive choices, newborns born in SCD prevalent regions of the world should be tested for SC traits to give caregivers an idea of how to care for children who may have SCD [4, 12, 13]. This care will include the medical attention that caregivers seek for their children.

Knowledge of parents and caregivers of SCD is important in the disease outcomes. Optimum knowledge is required for good management practices. In this study, more than half (60%) of the caregivers had poor knowledge of SCD. This is in agreement with the study by Armour and Jumanne [33] where most participants (53.3%) had inadequate knowledge about SCD. The finding of poor knowledge is also similar to the study among caregivers of children affected with SCD in Sudan [34] and Zambia [35] who reported that 46.9% and 53.9% had poor knowledge respectively among the caregivers. A study in Northern Ghana that explored the knowledge of caregivers of children with SCD, reported that caregivers had adequate knowledge of the signs and symptoms of SCD, its complications, and the various types of SCD but had poor knowledge on the cause of SCD. The difference in knowledge could be explained by the study type and methods of knowledge assessment. In general, knowledge of sickle cell disease has been reported to be poor in Ghana [36, 37].

Research suggests that knowledge of best health practices, including nutrition knowledge, can help reduce hospitalization and health care cost among patients with SCD [17]. In this study, despite most of the caregivers agreeing that certain health practices including adequate diet were important in disease management, the majority had poor nutritional knowledge regarding SCD management. A few of the caregivers considered nutrition care when their ward experienced a crisis. Islam et al., [38] corroborates this finding in their study where only a few home-based caregivers considered diet during SCD episodes even though they believed that diet played a role in their ward’s health. The nutritional implication of SCD has a direct consequence on the pathophysiology of the disease process, and it is often underused and poorly understood. The poor nutrition-related knowledge of SCD among the caregivers could be the reason less than a quarter of the caregivers in this study considered nutritional care during crises of their children. In Ghana, and many African countries nutritional care is not well-integrated into the care of patients with SCD, and patients are often referred for nutrition therapy after worse outcomes. It is important for caregivers to understand the complexity of SCD management including nutritional support to help improve growth and development, reduce chronic anaemia, promote weight maintenance, conserve muscle mass, and reduce inflammation for these patients.

The few caregivers in this study who considered nutrition support during crisis indicated providing their wards with more fruits, warm fluids and/or dark green leafy vegetables. Similar to the present study, Ajinkpang and colleagues [3] reported that caregivers often opted to feed their wards with regular family meals when they were in crisis. In that study, a few caregivers applied the concept of trying to provide more iron-rich foods as observed in the current study, mainly because health care providers informed them. In another study by Olwit and colleagues [23], caregivers did whatever they could (i.e. gave medication, meals, warmth) to help alleviate their ward’s pain during crises. Doing these things gave them a sense of relief to know that their ward was not in pain any longer. It is possible that caregivers provide these items because they consider them to be good sources of specific nutrients and factors that are perceived to reduce painful episodes. There is however, limited studies that have shown that acute SCD pain is reduced by nutritional interventions. Generally, children with SCD have inadequate levels of macro and micronutrients [14, 18], making them nutritionally vulnerable compared to their otherwise healthy counterparts. Regarding nutrition-related attitudes, most caregivers were unsatisfied with the eating habits of their wards who have SCD but perceived their wards to be of good nutritional status. The nutritional status of children is affected by a myriad of factors including diet, ongoing illness, genetics, and their environment [1, 21, 39]. In this study, diet was the predominant factor identified by caregivers as contributing to their wards’ nutritional status, however, some of the caregivers reported not knowing what led to their wards’ current nutritional status. Ajinkpang and colleagues [3] found comparable results. Their study reported high level of awareness and usage of ‘special foods’ specifically for SCD. Although health providers encouraged their intake, these ‘special foods’ did not help improve the condition of their wards. Owoo and Tadros [40] found that mothers of children with SCD would provide certain foods to their children due to their awareness of their ward’s nutrition needs. Knowledge of food selection and combinations is critical as it has an impact on the nutritional status of a child with SCD [20]. An understanding of the role nutrients such as iron and folate play in the life cycle of red blood cells and consistent application of appropriate dietary practices for children with SCD produce the most positive results as compared to intervening during crisis solely [17–19, 21].

Owoo and Tadros [40] acknowledged that to manage SCD among children effectively, parents need to have more knowledge about the condition than the average person. The few caregivers with high knowledge of SCD in this present study agreed that food could improve the health of people with SCD, and they reported that their wards did not experience crisis often, as compared to caregivers with low knowledge about SCD. Similar to this present study, Aderotoye-Oni and colleagues [12] found that knowledge about SCD increased as level of formal education rose among caregivers. This corroborates the suggestion to provide public messages on SCD at different literacy levels. As pointed out by Aderotoye-Oni et al., [12], Nnodu et al., [41], and Kilonzi et al., [42], there may be a need to tailor SCD educative materials to different caregivers’ information needs in order to assist home-based caregivers to tend to their wards with SCD.

In this study, caregivers did not struggle to talk about their wards’ condition with people in general, a possible indication that SCD is currently not heavily stigmatized in Ghana. The majority of interviewed caregivers were females, as well as the biological parent of the adolescent, and most reported overwhelming difficulty regarding the financial aspects of tending to a child with SCD. These observations suggest that caregivers of children with special health needs including SCD require additional economic and societal support especially in Sub-Saharan Africa [3, 23, 43, 44].

## Conclusion

This study found low level of knowledge of SCD among caregivers of adolescents with SCD as well as nutrition-related knowledge of the disease. Recognising the complex and multifactorial needs of SCD patients who are adolescents, there is the need to ensure comprehensive care at the health facility and home care by parents and caregivers of these children. At the facility level, comprehensive management of SCD needs to be consistent in incorporating nutrition counselling for parents/caregivers to improve their knowledge of the disease, related complications and recommended home-based practices to reduce the occurrence of complications and hospitalization.

## Electronic supplementary material

Below is the link to the electronic supplementary material.

Supplementary Material 1

Supplementary Material 2

## Data Availability

The datasets generated and/or analysed during the current study are not publicly available because it is part of a larger study that is yet to be completed and published but are available from the corresponding author on agreement with the co-authors upon reasonable request.

## Abbreviations

SCD: Sickle Cell Disease
SCDC: Sickle Cell Disease Clinic
ECBAS: Ethics Committee for the College of Basic and Applied Science
SPSS: Statistical package for social scientists
NCD: Non communicable diseases
WHO: World Health Organisation
UNICEF: United Nations International Children’s Emergency Fund
