# Supplementary material for: Knowledge and nutrition-related practices among caregivers of adolescents with sickle cell disease in the Greater Accra region of Ghana
Source: BMC Public Health. 2023 Mar 6;23:434. doi: 10.1186/s12889-023-15343-1 (PMC9990343; doi:10.1186/s12889-023-15343-1)
Supplement: Supplementary file 1 — Supplementary Material 1 [file 12889_2023_15343_MOESM1_ESM.docx]

Supplementary Table 1: Comparison of caregiver characteristics based on knowledge

|  | General SCD knowledge^#^ | | | | P-value^a^ | Nutrition-related knowledge^#^ | | | | p-value^a^ |
| --- | --- | --- | --- | --- | --- | --- | --- | --- | --- | --- |
|  | **Low**  **(n = 135)** | | **High**  **(n = 90)** | |  | **Low**  **(n = 159)** | | **High**  **(n = 66)** | |  |
| Sex:  Male  Female | 21  114 | 50.0  62.3 | 21  69 | 50.0  37.7 | 0.142 | 24  135 | 57.1  73.8 | 18  48 | 42.9  26.2 | 0.033 |
| Formal education:  < Secondary  ≥ Secondary | 67  68 | 78.8  48.6 | 18  72 | 21.2  51.4 | < 0.001 | 74  85 | 87.1  60.7 | 11  55 | 12.9  39.3 | < 0.001 |
| ^#^ Level of general knowledge about sickle cell disease was categorized using the group mean as cut-off: caregivers with values above 2.6 were classified as having high knowledge, those with values below 2.6 were classified as having low knowledge. Nutrition-related knowledge was based on only the five questions related to nutrition. The total score was categorized using the group mean of 1 as cut off: caregivers with values 1 and above were classified as having good nutrition-related knowledge, those who scored zero were classified as having low knowledge.  ^a^Analysis based on Pearson’s chi Square test. | | | | | | | | | | |
