# Supplementary material for: Knowledge and nutrition-related practices among caregivers of adolescents with sickle cell disease in the Greater Accra region of Ghana
Source: BMC Public Health. 2023 Mar 6;23:434. doi: 10.1186/s12889-023-15343-1 (PMC9990343; doi:10.1186/s12889-023-15343-1)
Supplement: Supplementary file 2 — Supplementary Material 2 [file 12889_2023_15343_MOESM2_ESM.docx]

Supplementary Table 2: Association between caregiver demographic characteristics and SCD-related knowledge, based on logistic regression model

| Variable | High general knowledge | | | High nutrition-related knowledge | | |
| --- | --- | --- | --- | --- | --- | --- |
|  | OR | 95% CI | p-value | OR | 95% CI | p-value |
| **Age**  < 42 years  ≥ 42 years | 0.958  1 | 0.538 -1.706 | 0.884 | 1.075  1 | 0.577 - 2.001 | 0.820 |
| **Sex**  Female  Male | 0.972  1 | 0.466 - 2.029 | 0.940 | 0.710  1 | 0.335-1.507 | 0.373 |
| **Level of Education**  ≥ Secondary  < Secondary | 3.900  1 | 2.055 - 7.403 | <0.001 | 4.030  1 | 1.920-8.457 | <0.001 |
| Odds ratio was obtained using binary logistic regression. Hosmer – Lemeshow Statistic: P = 0.05 for the ‘general knowledge’ model, p = 0.430 for the ‘nutrition-related knowledge’ model. | | | | | | |
